# Public Information Needs and Interest in Specific Food and Drug Allergy Disorders in Germany (2022–2024): Google Search Engine Analysis

**DOI:** 10.2196/75395

**Published:** 2025-11-12

**Authors:** Tobias Fuchs, Michael Hindelang, Sebastian Sitaru, Alexander Zink, Julia Welzel

**Affiliations:** 1 Department of Dermatology and Allergy, TUM School of Medicine and Health, Technical University of Munich, Biedersteiner Str. 29, Munich, 80802, Germany, 49 1606159509; 2 Pettenkofer School of Public Health, Munich, Germany; 3 Institute for Medical Information Processing, Biometry and Epidemiology (IBE), Faculty of Medicine, Ludwig-Maximilian University, LMU Munich, Munich, Germany; 4 Department of Dermatology and Allergology, University Hospital Augsburg, Augsburg, Germany

**Keywords:** food allergies, drug allergies, public health informatics, web search analysis, digital health literacy

## Abstract

**Background:**

The prevalence of food and drug allergies has been steadily increasing in Germany. These conditions not only impair the quality of life of those affected but also place an additional burden on the health care system. At the same time, an increasing number of people are using the internet and other digital sources to seek health-related information.

**Objective:**

This study aimed to use the Google Ads Keyword Planner to identify the information needs and knowledge gaps of the internet-using population in Germany and to provide a foundation for future prevention and educational strategies regarding food and drug allergies.

**Methods:**

Relevant keywords related to selected food and drug allergies were extracted using the Google Ads Keyword Planner and analyzed according to predefined criteria. The observation period was from September 2022 to October 2024.

**Results:**

A total of 633 keywords related to specific types of food and drug allergies were identified, generating a combined search volume of 3,649,390 queries. The most frequently searched terms nationwide were “histamine allergy” (368,980/3,649,390, 10.1%), “penicillin allergy” (266,410/3,649,390, 7.3%), and “nut allergy” (103,850/3,649,390, 2.8%). Although “histamine allergy” was the most frequently searched term in this analysis, most searches for “histamine allergy” likely referred to an intolerance rather than a true immunoglobulin E–mediated allergy. Seasonal patterns were also observed, with increased searches for the categories “nut” and “penicillin” in the winter months and for “histamine” in the spring months.

**Conclusions:**

This study demonstrates the potential of Google search query data analysis in a medical context and, in particular, underscores its relevance for understanding the public interest in food and drug allergies in Germany. The findings highlight the need for improved, easily accessible educational resources and for implementing allergy-specific, socially relevant health campaigns to address the unmet information needs of the population living in Germany regarding food and drug allergies.

## Introduction

### Background

The global prevalence of food allergies has been steadily increasing since the early 21st century [1-4], making them a widespread public health concern. It is estimated that at least 2% to 4% of all children and adults are affected [5,6], with current projections suggesting that approximately 520 million individuals worldwide have food allergies. This corresponds to a prevalence of 3% to 10% among children and up to 10% among adults [7]. The prevalence of drug allergies is of a similar magnitude. Self-reported data indicate that 7% to 8% of the general population has a medication allergy, and approximately 15% of all hospitalized patients are reportedly allergic to beta-lactam antibiotics [8-10]. These allergies not only impair the quality of life of those affected and their families [11] but also impose substantial costs on health care systems [12].

In Germany, the prevalence of allergic diseases has also risen steadily since the 1970s. According to the German Gesundheit in Deutschland aktuell 2019-2020–European Health Interview Survey study conducted between April 2019 and September 2020, among respondents, 31% reported having an allergic disease, excluding allergic asthma [13,14]. Women were found to be more frequently affected than men [13]. Data from the Commission for Environmental Medicine of the Robert Koch Institute indicate that the lifetime prevalence of food allergies among adults in Germany is 4.7% [15]. According to the most recent guideline, the prevalence is estimated at 3.7% [16], with common triggers including nuts, soy, and dairy products [16]. Reliable data on the prevalence of drug allergies in Germany remain scarce. However, point prevalence rates for penicillin allergy—one of the most important drug allergies—were reported at 17% in both 2019 and 2022 at the University Medical Center Hamburg-Eppendorf [17]. Both food and drug allergies can cause mild symptoms, such as urticaria or exanthema, as well as potentially life-threatening anaphylactic reactions [18].

The prevalence figures and the potential severity of symptoms underscore the public health relevance of these types of allergies and the urgency of addressing them—at both the clinical and patient levels.

However, the sometimes fluctuating data also highlight the challenges in accurately determining the true number of individuals affected. Reported figures are often based solely on patients’ self-reported histories or on general population surveys. The already complex medical differentiation between a confirmed allergy, an intolerance reaction, and an adverse drug reaction is further complicated by the growing influence of digital nutrition “experts,” lifestyle coaches, and self-help groups providing lifestyle advice. As a result, patients may assume that they have certain allergies and avoid the relevant allergens—with all associated consequences—even when allergological diagnostics fail to confirm the suspected allergy [19]. The goal should be to ensure effective treatment for individuals who are truly affected through early and targeted diagnostics, while simultaneously promoting consistent “delabeling” of nonexistent allergies [17]. Furthermore, public awareness of this issue in general and the clear differentiation between allergic and nonallergic reactions in particular should be strengthened through improved public health education.

In addition, with the ongoing digitalization of the health care sector and the expanding range of telemedical information sources, patients can now independently access information about their illnesses and symptoms online, without relying solely on medical professionals. This trend has been further accelerated by the COVID-19 pandemic, and the public’s willingness to seek health information digitally continues to grow [20].

### Objectives

To better understand and contextualize the current level of knowledge and the unmet information needs of the German population regarding food and drug allergies, a Google search query analysis was conducted as part of this study. The results are presented in detail herein.

A Google search query analysis is a method in which user-generated search query data are collected using tools such as the Google Ads Keyword Planner and then systematically analyzed. Initially, this approach was primarily used to optimize corporate advertising campaigns [21]. However, because this form of web analysis can reflect the various interests of the internet-using population, it has increasingly been adopted in scientific research [22-25]. Topics relevant to allergic diseases have also been analyzed with this tool [26-29]. For the first time, to the best of our knowledge, this study applies this method to the context of food and drug allergies. In Germany, such analyses are particularly effective when conducted via the Google search engine, which remains the market leader with a share of 88.2% as of November 2024 [30], making it the primary platform for the majority of the country’s internet users. Overall, 94% of Germany’s total population—nearly 80 million people—currently use the internet [31,32]. Therefore, a Google-based analysis can capture data reflecting the search behavior of a substantial proportion of the German population.

## Methods

### Overview

This Google-based analysis was conducted as a scientific extension of an ongoing research project evaluating the benefits of the What’s in My Meds medication app for patients with allergies. Accordingly, the same food and drug allergies included as selectable options in the What’s in My Meds smartphone app were considered in this analysis. The geographic focus was similarly aligned, targeting the Munich and Augsburg regions. This complementary project enables a meaningful supplementation of the app-based data, thereby enhancing the specificity and strengthening the relevance of both studies within a comparable regional context.

The Google Ads Keyword Planner was used to analyze search volumes in the field of allergies. Search volume refers to the number of search queries generated by internet users for a specific term (keyword) [21]. The Keyword Planner generates a list of search terms related to predefined topics relevant to the study. These search terms may consist of single words or context-related phrases entered by users in the Google search engine [20]. In addition, the Keyword Planner provides an estimate of the average monthly search volume for each relevant term. Collected data can be retrieved retrospectively for up to 48 months from the time of the query [20].

### Statistics

This study primarily analyzed search queries related to allergies to selected medications and foods, as well as seasonal and regional variations in the most frequently searched keywords. Keyword data were retrieved using the Google Ads Keyword Planner application programming interface under a registered institutional account. After retrieval, an initial screening of the keywords was conducted, during which their clinical and contextual relevance was discussed within the research team. Final keyword inclusion was determined by consensus.

The analysis considered allergies to beta-lactam antibiotics (eg, penicillins, aminopenicillins, and cephalosporins), analgesics (eg, aspirin, ibuprofen, metamizole, and diclofenac), histamine, nuts, soy, egg proteins (albumin and lecithin), macrogol, and parabens. These allergens were selected because they corresponded to those examined in the related What’s in My Meds app study. In addition to the allergy terms themselves, search queries regarding allergy-related symptoms or potential cross-allergies were included.

While the analysis covered the whole of Germany, a particular focus was placed on the southern regions, especially Munich and Augsburg. Keywords in German were primarily examined, although relevant English terms were also included. The evaluation period spanned from September 2022 to October 2024.

The data were analyzed descriptively, and the most frequent keywords were qualitatively categorized. This categorization into thematic groups (“histamine,” “nuts,” “soy,” and “penicillin”) was performed by the first author and independently reviewed for consistency and completeness by other members of the study team. Data analysis was conducted using SPSS software (version 29.0; IBM Corp).

## Results

### Overview

A total of 633 distinct keywords related to “food allergy” and “drug allergy” were identified, generating a combined search volume of 3,649,390 queries during the observation period from September 2022 to October 2024. Distributed by year, the 3,649,390 queries corresponded to 489,630 (13.4%) in 2022, a total of 1,666,690 (45.7%) in 2023, and 1,493,070 (40.9%) in 2024. The lower figure for 2022 is explained by the fact that only data from September to December were included, whereas 2023 and 2024 were fully or nearly fully represented. When the 3,649,390 search queries were broadly assigned to the categories “food allergy” and “drug allergy,” the respective volumes were 3,380,720 (92.6%) and 268,670 (7.4%), indicating that queries about food allergies accounted for a vast majority of all searches. Regarding language, 3,567,950 (97.8%) of the 3,649,390 searches were conducted in German and 81,440 (2.2%) in English. The 16 most frequently searched terms are presented in Table 1. In the Google AdWords Keyword Planner, synonyms (eg, “nut allergy” and “nut allergies”) are listed separately, even when they share identical search volumes; therefore, synonymous terms with identical counts were consolidated for this analysis. Across the entire observation period, the most frequently searched term was “histamine allergy” (368,980/3,649,390, 10.1%), followed by “penicillin allergy” (266,410/3,649,390, 7.3%) and “nut allergy” (103,850/3,649,390, 2.8%). It should be noted that searches for “histamine” likely reflect lay terminology that conflates histamine intolerance with histamine allergy, highlighting common public misunderstandings. Overall, the top 5 keywords accounted for 25.1% (917,070/3,649,390) of the total search volume. Allergy-related terms concerning analgesics were searched far less frequently, with “ibuprofen allergy” being the most common (18,900/3,649,390, 0.5%).

**Table 1.** Google users’ top keywords in Germany regarding specific food and drug allergies listed by search volume (September 2022-October 2024; N=3,649,390).

Keywords (a consolidation of synonymous search terms was performed)	Search volume, n (%)
Histamine allergy (IgE^a^ mediated)	368,980 (10.1)
Penicillin allergy	266,410 (7.3)
Nut allergy, nut allergies, nut allergy sufferers	103,850 (2.8)
Allergy to soy	92,680 (2.5)
Histamine intolerance symptoms (non-IgE mediated)	85,150 (2.3)
Nut allergy symptoms, symptoms of a nut allergy, symptoms of nut allergy, symptoms nut allergy	75,700 (2.1)
Soy allergy, allergy soy, allergens soy, soya allergy	46,340 (1.3)
Intolerance to soy, soy intolerance	39,680 (1.1)
Peanut allergy symptoms	33,330 (0.9)
Allergy to histamine (IgE mediated)	33,140 (0.9)
Hazelnut allergy, allergy hazelnuts, allergy to hazelnuts	32,740 (0.9)
Symptoms of histamine allergy, allergy histamines symptoms, allergy histamine symptoms, histamine allergy symptoms (IgE mediated)	31,170 (0.9)
Cross allergy hazelnuts	29,120 (0.8)
Walnut allergen	28,680 (0.8)
Allergy hazelnut	26,160 (0.7)
Allergy soy symptoms	24,470 (0.7)

a IgE: immunoglobulin E.

### Categorization of Content

In addition, the top 16 keywords were categorized by content. While search queries for these keywords were evaluated individually, a thematic aggregation was also performed. Accordingly, all search terms within the top 16 were grouped into the categories “nuts,” “histamine,” “soy,” and “penicillin.” The total search volumes for each thematic group were then analyzed. The resulting search volumes for the categorized top 16 keywords across the entire observation period are described in more detail below.

Searches related to “nuts” accounted for 20.1% (733,527/3,649,390) of the total search volume, followed by “histamine” (613,098/3,649,390, 16.8%), “soy” (383,186/3,649,390, 10.5%), and “penicillin” (266,405/3,649,390, 7.3%).

Search terms concerning analgesics were also categorized by content. The category “ibuprofen” accounted for 2.4% (86,820/3,649,390) of the total search volume, followed by “diclofenac” (8050/3,649,390, 0.2%), “acetylsalicylic acid” (2260/3,649,390, 0.1%), and “naproxen” (330/3,649,390, 0.0%).

### Regional Differences

**Figure 1.**
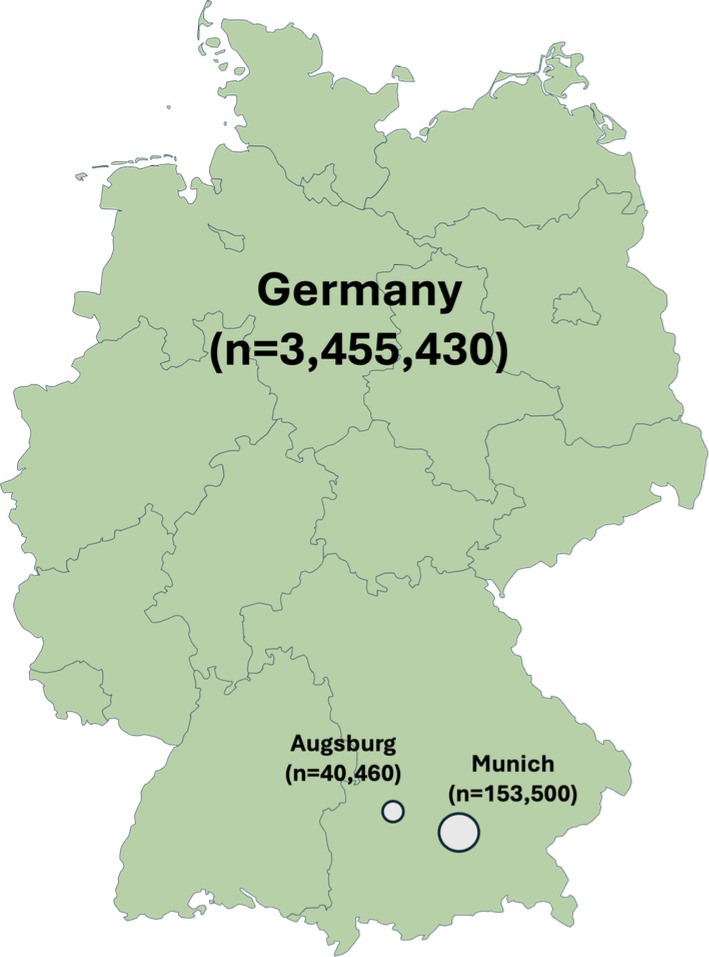
Regional distribution of Google search volumes regarding specific food and drug allergy search terms in Germany (September 2022-October 2024).

Of the 3,649,390 searches, 153,500 (4.2%) and 40,460 (1.1%) were recorded in Munich and Augsburg, respectively, over the entire observation period, whereas, for the rest of Germany, the search volume amounted to 3,455,430 (94.7%) queries (Figure 1).

The linguistic distribution of search queries in the respective regions was as follows: of the 3,455,430 search queries from the rest of Germany, 3,377,950 (92.6%) were in German and 77,480 (2.1%) in English. In Munich, of the 153,500 queries, 1,50,450 (98%) were in German and 3050 (2%) in English, while in Augsburg, of the 40,460 queries, 39,550 (97.8%) were in German and 910 (2.2%) in English.

A qualitative classification of all keywords into the main categories of “histamine,” “nuts,” “penicillin,” and “soy” was also performed to assess regional differences. All remaining search terms were grouped under the heading “Other.” Table 2 presents the regional differences in search behavior for the top 4 categories—expressed per 100,000 inhabitants—for Germany as a whole as well as Munich and Augsburg.

**Table 2.** Regional differences in Google search volumes for food and drug allergy categories, including the top 4 and “Other,” per 100,000 inhabitants in Germany (September 2022-October 2024).

Categories	Germany (except Munich and Augsburg)	Munich	Augsburg
Other	571.8	2066.9	3342.2
Histamine	1008.8	2451.3	2804.1
Nuts	1568.0	3541.9	5010.0
Penicillin	326.7	559.1	364.3
Soy	677.8	1695.3	2002.0

### Seasonal Trends

**Figure 2.**
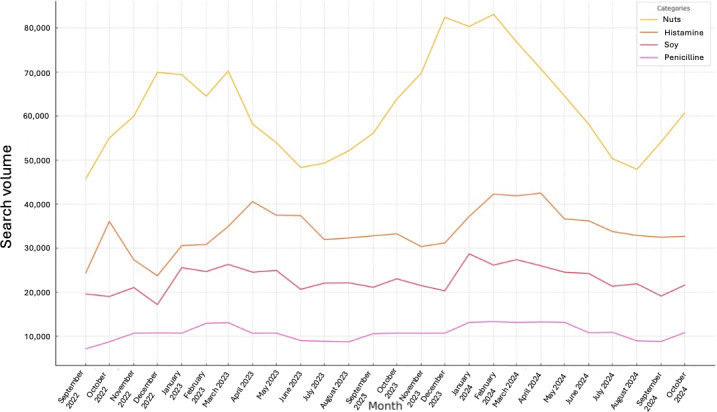
Monthly variation in Google users’ top 4 search categories regarding specific food and drug allergies in Germany (September 2022 to October 2024).

To provide an overview of seasonal trends, the most frequently searched categories over the entire study period were analyzed for all regions examined. These categories included all search queries related to “nuts,” “histamine,” “soy,” and “penicillin.” Figure 2 illustrates the monthly variation in search volumes for these top 4 categories throughout the observation period.

The seasonal distribution of search volumes for the categories “nuts,” “histamine,” “soy,” and “penicillin” revealed clear patterns and recurring fluctuations across 2022, 2023, and 2024. For “nuts,” an increase in search volume was consistently observed during the winter months of each year, peaking in February 2024 over the entire observation period. From April onward, search volumes declined sharply, reaching their lowest levels during the summer months, particularly between May and August. By contrast, the “histamine” category exhibited the opposite pattern: peak search volumes occurred mainly in March and April, followed by a steady decline continuing into the winter months. The only deviation from this trend was a secondary peak in October 2022, which cannot be fully contextualized due to the absence of data from earlier months that year. The “soy” category reached its highest search volumes in 2023 and 2024, especially between January and May, with a peak in January 2024. Overall, fluctuations in “soy” search volumes were less pronounced than those observed for “nuts” and “histamine.”

The “penicillin” category showed a moderate increase during the winter months of each study year, extending into May in 2024. From spring onward, search volumes gradually declined—sometimes with a slight delay—before stabilizing at a lower level during the summer months. These observed seasonal patterns were consistent across all years analyzed.

## Discussion

### Principal Findings

The Google-based analysis conducted in this study provides valuable insights into the search behavior and distribution of interest within the German population regarding “food allergy” and “drug allergy.” In the context of this study, the term “German population” refers to individuals living in Germany who use the internet, regardless of country of origin or citizenship. With a total of 3,649,390 search queries, it is evident that allergy-related topics are of substantial public interest and are regularly researched via the Google search engine. The distribution of search queries suggests that conditions such as nut allergy and histamine intolerance are subjects of active public discussion, potentially driving the observed high search volumes. However, this association requires validation through future studies, as current evidence is lacking. In the case of nut allergies, heightened interest may be linked to the potential severity of reactions—members of the nut family account for 70% to 90% of all food-related anaphylactic deaths [33]. The public relevance of histamine intolerance may be explained by the presence of histamine in commonly consumed foods and beverages such as cheese, fish, and red wine [34]. Notably, searches for “histamine” were often framed as “histamine allergy,” the single most searched term in the study period. Scientifically, such cases more likely represent intolerance reactions—caused by reduced histamine breakdown due to potential diamine oxidase deficiency—rather than true immunoglobulin E–mediated allergies [35]. The existence of histamine intolerance itself is still debated among experts [36,37]. These findings highlight the need for improved medical education, as the distinction between allergy and intolerance can have significant therapeutic and prognostic implications.

A similar situation applies to “penicillin allergy,” the second most frequently searched term. Although its public relevance is clear, the diagnosis should be approached with caution. Differentiating between adverse effects and true allergic reactions is challenging; for instance, aminopenicillin administration during an Epstein-Barr virus infection may trigger a rash resembling an allergy but without life-threatening implications [38]. Many older patients report penicillin allergies dating back to childhood and, consequently, avoid all derivatives. This avoidance can be problematic, as penicillins remain the first-line therapy for many bacterial infections. Uncritical acceptance of self-reported allergies often leads to the use of broad-spectrum reserve antibiotics, further exacerbating antimicrobial resistance [39]. Given the public interest indicated by our findings, there is a strong need to raise awareness in clinical practice and to promote systematic “delabeling” of inaccurately assigned allergies [17]. By contrast, searches for allergic reactions to analgesics were far less frequent, despite being among the most common drug-induced causes of anaphylaxis [40]. This may reflect a general lack of public awareness of analgesics as potential allergens, lower perceived social relevance, or the fact that painkillers are typically used intermittently or by specific patient groups (eg, patients with chronic pain or those with cancer) [41]. Older adults, who often require analgesics for rheumatic or degenerative diseases [42], are also less likely to seek information online, limiting the representativeness of Google search data for this topic. Nonetheless, health care providers should increase public awareness of analgesics as possible allergens, with family physicians ideally leading such efforts, as they are often the first point of contact for patients and have the most comprehensive overview of their long-term and as-needed medications. Educational strategies could include printed information materials, short instructional videos, and other outreach initiatives, as described later in this paper. Seasonal analysis revealed distinct trends. The winter peak in nut allergy searches may relate to increased consumption of nuts during the Christmas period, while the spring and summer rise in histamine-related searches could reflect the seasonal availability of histamine-rich foods such as strawberries and citrus fruits. The winter increase in penicillin allergy searches aligns with the documented patterns of antibiotic overprescription in colder months [43]. At present, these seasonal interpretations remain hypotheses, as the available data are limited, underscoring the need for further investigation.

The regional analysis revealed notable differences. As expected, Munich had a higher total search volume than Augsburg, which can be attributed to its larger population. When comparing the search volumes for the top 4 categories per 100,000 inhabitants in Munich and Augsburg with those for Germany overall, it becomes evident that urban search volumes are substantially higher than the national average. One possible explanation is greater medical awareness in cities due to higher educational levels or more direct access to digital media compared with rural areas. To support this hypothesis, future studies should compare data across federal states and include other major German cities. Nevertheless, it remains unclear to what extent factors such as educational level, cultural differences, or access to medical information influenced the results, as such aspects are difficult to assess through search engine analysis alone.

The methodology applied—particularly the use of the Google Ads Keyword Planner—allowed for systematic recording and categorization of search behavior but also carries limitations. First, the analysis includes only searches conducted via Google. While Google remained the market leader in Germany in 2024 with a market share of 88.2%, other search engines still account for a portion of the market. Consequently, the search behavior of approximately 10 million people is not represented. Second, it is questionable whether the analyzed queries are fully representative of the entire German population, as groups considered vulnerable and with limited digital engagement—such as older adults or rural populations with restricted internet access—may be underrepresented. According to an annual online study conducted by German public broadcasters in 2022, internet use declines significantly among those aged more than 70 years [44]. Given that this age group comprises approximately 15 million people, or roughly 19% of the German population [45], this likely represents a major limitation of the study. It also remains unclear whether the analyzed search queries were generated by individuals personally affected, by professionals, or on behalf of others (eg, parents searching for information for their children). As children are frequently affected by food allergies [2], it is plausible that a proportion of the relevant searches originated from parents or guardians. In addition, while keyword categorization was essential for analysis, it risks oversimplifying the data and overlooking nuanced or highly specific issues.

Despite these limitations, the results indicate a substantial public demand for information about food and drug allergies via online search platforms. Further research is needed to determine whether this demand will continue to grow. As patients increasingly seek information online before consulting a health care professional [20], it is critical that search engine providers present medically accurate and scientifically sound content. At the same time, the medical community must provide evidence-based, patient-oriented information to strengthen health literacy and enable individuals to critically evaluate online medical resources—especially in the context of digital infodemics [46].

This study highlights the need to improve the public’s ability to distinguish between true allergies and intolerances and to encourage consistent “delabeling” of nonexistent allergies, such as inaccurately reported penicillin allergies. It also underscores the importance of raising awareness about other potent allergens, such as analgesics. Multiple strategies could be used to address these needs; for example, targeted educational campaigns in hospitals and outpatient clinics could be aligned with frequently searched terms and seasonal patterns, providing printed materials or posters focusing on specific allergens—for instance, nut allergies in winter or histamine intolerance in spring. Media campaigns involving collaborations between clinics, medical practices, health insurers, and allergy associations, as well as organizing allergy awareness events, could further increase public engagement. Establishing official allergy awareness days might also enhance visibility. Finally, expanding the range of digital information tools—such as regular use of smartphone apps or short educational videos on platforms such as Instagram Reels or TikTok—could help disseminate allergy-related knowledge to the general public, independent of direct physician contact. The key to these initiatives lies in balancing individual needs with societal priorities and in addressing all population groups and generations as inclusively as possible. If implemented strategically, these measures could sustainably improve awareness and understanding of food and drug allergies in Germany.

### Conclusions

The findings of this study underscore the considerable value of Google search query analysis for identifying the information needs of the German population regarding food and drug allergies. Such data can serve as a foundation for developing improved, evidence-based educational strategies and enhancing public awareness of the allergological topics highlighted in this study. The results demonstrate that individuals with allergies commonly use internet search engines such as Google to seek health-related information. Building on this, the future expansion of digital information tools—such as social media platforms, smartphone apps, or wearable technologies—could further support patients with allergies.

The analysis also revealed that histamine intolerance is still frequently perceived by the German public as a manifest allergy and that “penicillin allergy” and “nut allergy” remain highly prominent topics in public discourse.

Consequently, incorporating search engine analyses into health care research is advisable, both to gain an overview of public interest in specific health topics and to use the resulting data as a valuable complement to traditional epidemiological studies.

The informative value of such data could be further enhanced by integrating additional insights from social media analyses or qualitative interviews on specific issues. Supplementary studies addressing demographic and socioeconomic factors could deepen the analysis and help reduce potential biases in the research findings.
